# Probabilistic double guarantee kidnapping detection in SLAM

**DOI:** 10.1186/s40638-016-0053-z

**Published:** 2016-11-24

**Authors:** Yang Tian, Shugen Ma

**Affiliations:** 1 Department of Robotics, Ritsumeikan University, Shiga, 525-8577 Japan; 2 Department of Electrical Engineering and Automation, Tianjin University, Tianjin, 300072 China

**Keywords:** Kidnapping detection, Simultaneous localization and mapping, Autonomous mobile robots

## Abstract

For determining whether kidnapping has happened and which type of kidnapping it is while a robot performs autonomous tasks in an unknown environment, a double guarantee kidnapping detection (DGKD) method has been proposed. The good performance of DGKD in a relative small environment is shown. However, a limitation of DGKD is found in a large-scale environment by our recent work. In order to increase the adaptability of DGKD in a large-scale environment, an improved method called probabilistic double guarantee kidnapping detection is proposed in this paper to combine probability of features’ positions and the robot’s posture. Simulation results demonstrate the validity and accuracy of the proposed method.

## Background

Different fields like factories, hospitals and houses require mobile robots to navigate autonomously and to perform tasks by themselves. In this situation, robots should be able to make a map of the environment and recognize its posture (position and orientation) in this map [1–3]. Simultaneous localization and mapping (SLAM) is a fundamental technique that can provide the required information to mobile robots [4, 5]. In SLAM, a robot incrementally builds a consistent map of the environment while simultaneously determining its posture within this map. Many algorithms have been proposed to be implemented in a number of different autonomous mobile robots ranging from indoor and outdoor robots to underwater and airborne vehicles. The methods existing today allow the problem to be considered as solved, but some issues still need to be studied.

Some SLAM methods using current sensor and odometry data are based on pose tracking, which is a localization method for detecting the location of the mobile robot based on a given initial robot posture. Starting from this point, the robot posture is recognized by continuously tracking the robot’s path. If a well-tracked robot is suddenly moved to somewhere else without being told, the problem is called kidnapping. The autonomous robot should detect this problem in real time; otherwise, failures or faults are caused by the effects from kidnapping. Thus, the robot may execute a wrong action using the wrong information, even hurting humans, environment or damaging itself.

**Fig. 1:**
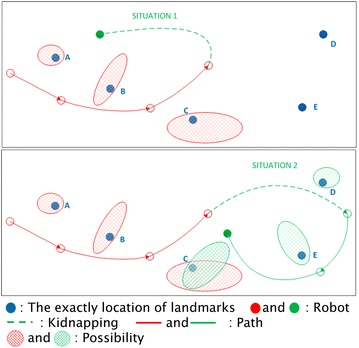
Two kidnapping situations in SLAM which includes the robot kidnapped to explored area or unexplored area

Especially in SLAM process, there are two different types of situations that may occur if kidnapping happens, which are shown in Fig. 1. When the mobile robot is doing the SLAM process, the environment will be mapped with features. If kidnapping happened, like in situation 1, the mobile robot will be kidnapped to a previously explored area. Then, the mobile robot can recognize its posture in the global coordinates with the known map which was created by SLAM. In situation 2, since the robot is kidnapped to a new area, the true location cannot be estimated by the existing map. The mobile robot thus needs to create a new SLAM process to estimate its position in a new global coordinates. Either situation needs kidnapping detection to identify whether kidnapping has happened, because the existing kidnapping recovery method is impossible to be executed all the time if the kidnapping belongs to situation 2.

**Fig. 2:**
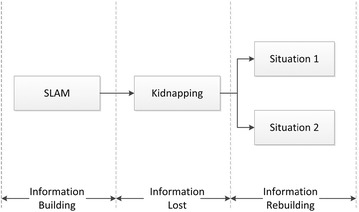
Information state during kidnapping in SLAM. When the SLAM is performing, the information about the robot and the environment is building; since the kidnapping will cause the information incorrect, correct information should be rebuilt by suitable methods with different situations

While kidnapping happens in SLAM, the original information including states of the robot and the map generated by SLAM will be affected without a kidnapping detection, as shown in Fig. 2. First, the robot creates the extra incorrect information, such as a wrong position of the robot and new features. Since this incorrect information cannot refer to original global coordinates, the mobile robot cannot utilize this information to navigate autonomously. Second, with some classic SLAM algorithms such as EKF-SLAM [6] and FastSLAM [7, 8], the original information built before kidnapping is also deformed. This is the point that is different in known map kidnapping problem. Since the state of posture of robot and all features are correlated, there is influence on all information with the incorrect information. Therefore, the original information will be totally deformed without a timely kidnapping detection. For recovering from kidnapping, the mobile robot should rebuild information to locate itself. In this case, the efficiency of SLAM would be significantly reduced, especially in a large-scale environment.

In our previous research [9], we have proposed a double guarantee kidnapping detection (DGKD) in SLAM. It constructs a double guarantee to judge whether kidnapping has happened and which type of kidnapping it is. However, DGKD has its own limitation in the large-scale environment. In this paper, an improved method ‘probabilistic double guarantee kidnapping detection (P-DGKD)' is proposed to maintain similar ability of kidnapping detection in the large-scale environment. In the next section, the related work and limitation of DGKD will be described.

**Fig. 3:**
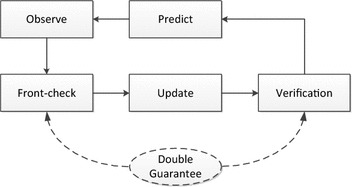
Overall workflow of the DGKD. It embeds two new processes in the ordinary SLAM processes to construct double guarantee

**Fig. 4:**
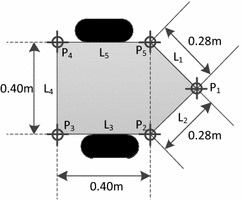
Dimensions of the robot

## Related work

Most of the literature focusing on the pose estimation has concentrated on the pose tracking problem. Some approaches explicitly dealing with sensing, model and movement uncertainty have appeared [10]. Common to these approaches is that they use a probabilistic formulation to represent and update the pose of the robot. This has the advantage of enabling them to handle uncertainty in a natural and convenient manner. These approaches, also known as Markovian methods, use a spatially discretized representation of the environment where each cell holds the probability that the robot occupies the area represented by the cell. They use a two-step procedure to update this representation, namely (using their nomenclature) the ‘move’ step where the fact that the robot moves is accounted for by shifting probability mass between cells according to the robot movement, and the ‘sense’ step where Bayesian updating is used to incorporate new evidence stemming from a feature/map comparison. In general, the ‘sense’ step concentrates probability mass in some areas and the ‘move’ step disperses it. This 'blurring' is due to the fact that the probability mass is not only shifted, but also smeared to account for robot movement inaccuracies. For the global localization to 'converge,' it is important that the evidence achieved in the ‘sense’ step more than compensates for the additional pose uncertainty introduced by the ‘move’ step. This fact stresses the importance of having an efficient movement/sensing strategy, i.e., to do active sensing, since moving randomly in general does guarantee gaining evidence efficiently enough. However, they do the re-localization (global localization) all the time, regardless of whether kidnapping has happened or not. For maintaining validity, these methods need to be executed at high frequency. It results in wasting lots of computational cost, which is inefficient and is not well suited for SLAM.

**Fig. 5:**
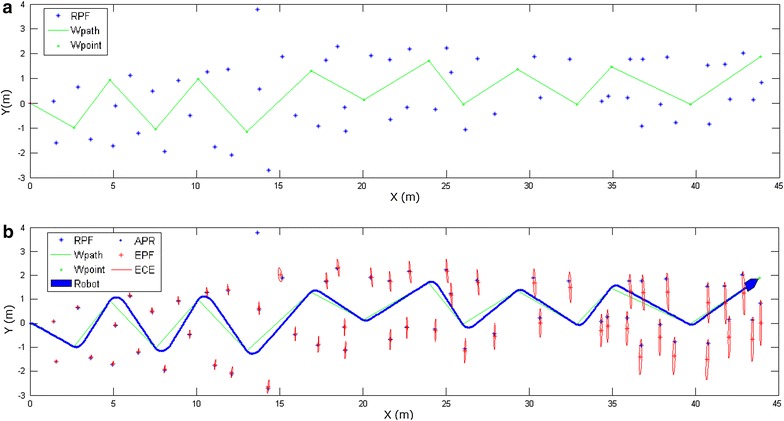
Map and EKF-SLAM. **a** Map for simulation. **b** Result of EKF-SLAM without kidnapping

**Fig. 6:**
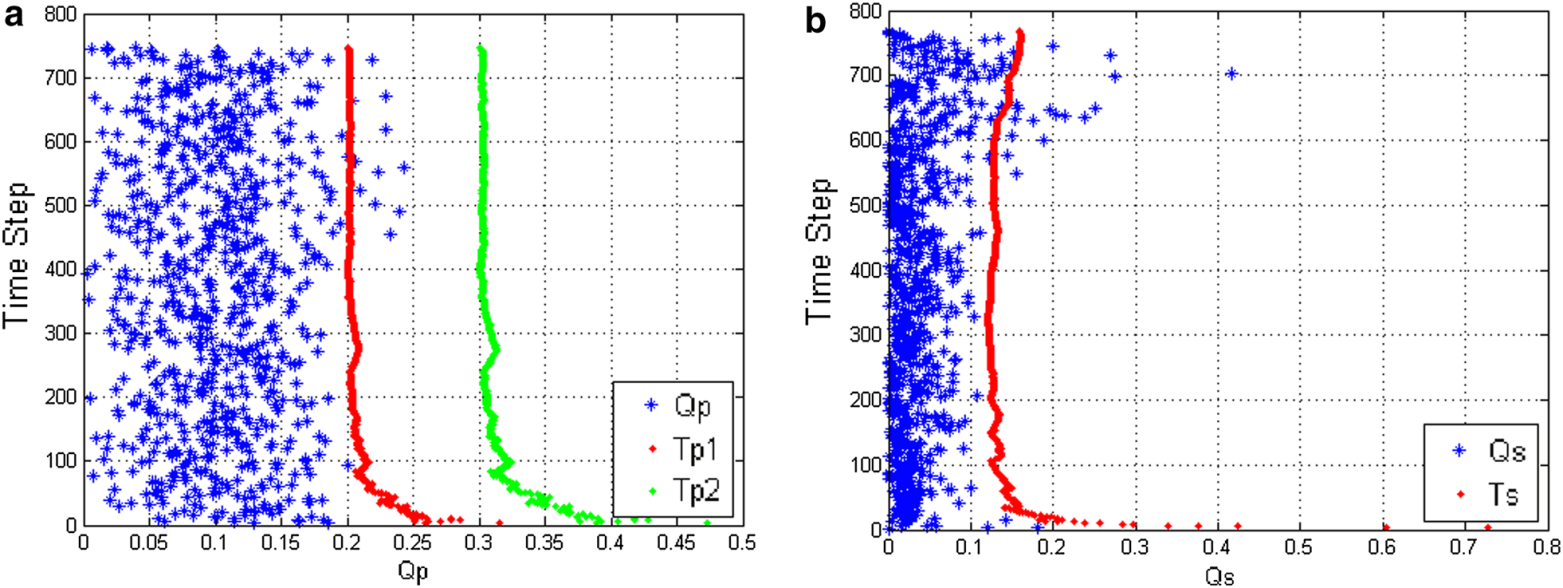
Non-kidnapping with DGKD. **a** Response of the metric $Q_p$. **b** Response of the metric $Q_s$

**Fig. 7:**
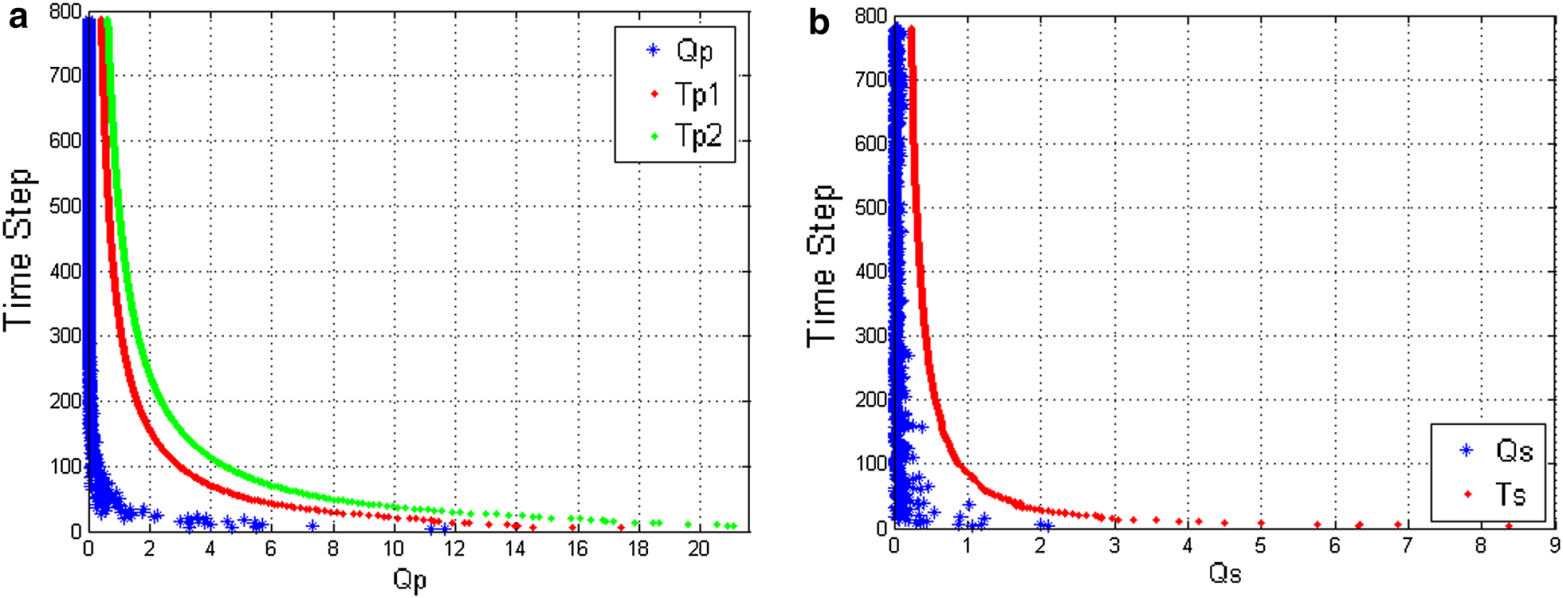
Non-kidnapping with P-DGKD. **a** Response of the metric $Q_p$. **b** Response of the metric $Q_s$

**Fig. 8:**
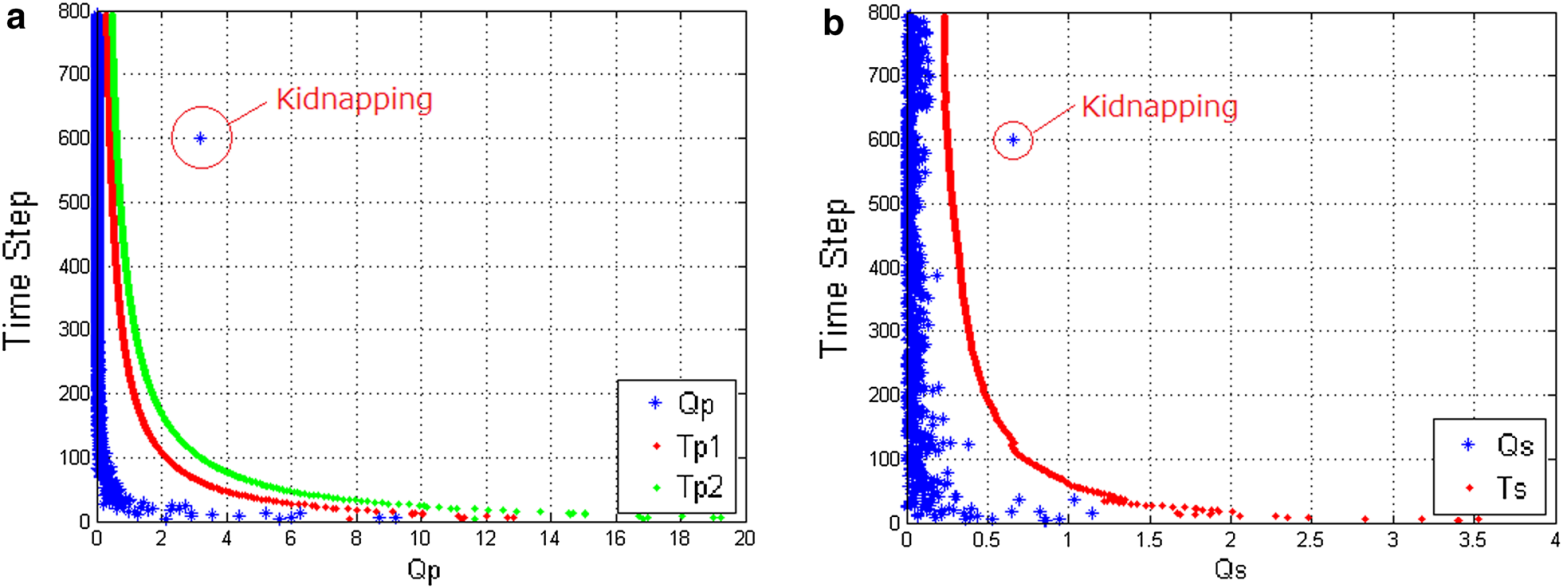
Kidnapping with P-DGKD. **a** Response of the metric $Q_p$. **b** Response of the metric $Q_s$

On the other hand, some efficient methods to solve the kidnapping detection have been proposed recently. Physical methods that use incorporate sensors to measure whether kidnapping has happened or not can be applied simply and directly, e.g., barometer [11], accelerometer [12] and switch [13]. However, there are limitations existing in these methods. First, additional sensors are required to be mounted on the robot. Therefore, the reliability of these methods cannot be ensured while the sensors’ state is abnormal. Second, each of the sensors can detect only a specific type of kidnapping. Third, the robots equipped with low power source cannot adopt these methods. Mathematical methods only utilize inherent sensors to observe abnormal situations. Compared to the physical methods, they can be used in the robots that have proprioceptive and exteroceptive sensors to locate themselves. Using entropy of location probabilities [14], the robot can detect kidnapping with the given information. However, it cannot be applied in SLAM where the information of the map is unknown. Metric-based detection [15] that can be utilized in unknown environment has a good performance in detecting the kidnapping. However, in some specific situations, it may fail to evaluate correctly if kidnapping has happened, such as the robot is kidnapped into a similar place in unexplored area.

**Table 1:** Simulation condition

Condition	Value
Robot speed	0.3 m/s
Control cycle	0.2 s
Observation cycle	0.2 s
Max range of observation	3 m
Variance of speed noise	0.09 m/s
Variance of orientation noise	9°
Variance of observation position noise	0.01 m
Variance of observation angle noise	1°

Comparing with the other studies, two new processes are added to execute the evaluation while SLAM is working in DGKD procedure. They have the double guarantees to judge whether kidnapping has happened and which type of kidnapping it is. Figure 3 shows overall workflow of DGKD. The method increases the reliability of kidnapping detection that prevents the information from deforming. In addition, a more reasonable metric has been introduced to avoid a misjudgment in a specific situation and a convenient method has been proposed to determine reasonable thresholds for the metrics on a real-time condition. However, DGKD has its own limitation in a relatively large-scale environment. To show the limitation of DGKD, simulation has been done in a relatively large-scale environment without kidnapping happening under the conditions shown in Table 1.

The size of the robot in our simulation is shown in Fig. 4. The map and non-kidnapping simulation are shown in Fig. 5. As indicated in Fig. 5a, ‘RPF’ denotes the real positions of the features. ‘Wpoint’ denotes the waypoint and ‘Wpath’ denotes the path connected with waypoints. The robot needs to drive itself toward each waypoint by the shortest path. The scale of the environment is relatively large according to the size of the robot and the range of the sensor. The scale of the map is about 11 times larger than the robot size and 15 times larger than the range of the sensor. Figure 5b shows the non-kidnapping SLAM progress with the map. ‘APR’ denotes the actual position of the robot. ‘EPF’ and ‘ECE’ represent the estimated position of the feature and its covariance ellipses. In non-kidnapping situation, the EPF is near the RPF and the distance between them becomes larger as time step passed. Figure 6 shows the response of metrics and their adapted thresholds. In non-kidnapping situation, the value of metrics $Q_p$ and $Q_s$ should be lower than the threshold $T_{p1}$ and $T_{s}$, where $Q_p$ and $Q_s$ belong to front-check process and verification process separately. However, some values of the metrics are beyond thresholds when time step increased, as shown in Fig. 6. This is because the uncertainty of the state has been increased by the process of SLAM, which is its own problem in the SLAM process. When the mobile robot is working in the large-scale environment without loop closure, the probability of false alarm is increased in DGKD by this increased uncertainty, which means that the total performance of DGKD is decreased. For keeping similar performance of DGKD in the large-scale environment, an improved method called probabilistic double guarantee kidnapping detection (P-DGKD) is described in the next section.

## Probabilistic double guarantee kidnapping detection

In this paper, we assume that the robot works in 2-dimensional space and the observation can be measured all the time. The robot’s state is described by the vector $X_r = [x_r, y_r, \phi _r]^T$, in which $(x_r, y_r)$ represents the position and $\phi _r$ represents the orientation of a frame attached to the robot. The state of features is denoted by $X_m = [X_{m1}^T, X_{m2}^T, \cdots ]^T$, in which $X_{mi} = [x_{mi}, y_{mi}]^T$ represents the position of the feature *i* in the global coordinates. $X_{mi}$ is given by

1 $$\begin{aligned} X_{mi} = \left[ \begin{array}{cc} \cos \phi _r &{} -\sin \phi _r \\ \sin \phi _r &{} \cos \phi _r \\ \end{array} \right] \left[ \begin{array}{c} ^Lx_i \\ ^Ly_i \\ \end{array} \right] + \left[ \begin{array}{c} x_r \\ y_r \\ \end{array} \right] \end{aligned}$$

where $(^Lx_i, ^Ly_i)$ represents the position of feature *i* referred to the local coordinates frame attached on the robot. Therefore, the state vector is $X = [X_r^T, X_m^T]^T$, which contains both the robot state $X_r$ and the feature states $X_m$.

In predicting process in SLAM, the predicted state $X(k+1|k) = [X_r^T(k+1|k), X_m^T(k+1|k)]^T$ at time steps *k* is given by

2 $$\begin{aligned} X(k+1|k) = F(X_r(k|k),u(k))+w(k) \end{aligned}$$

or

3 $$\begin{aligned} X(k+1|k) = \left[ \begin{array}{c} f(X_r(k|k),u(k)) \\ X_m(k|k) \\ \end{array} \right] + \left[ \begin{array}{c} w_r(k) \\ 0 \\ \end{array} \right] \end{aligned}$$

where $(X_r(k|k), X_m(k|k))$ is the state at the time step *k*, *u*(*k*) indicates the control measurement at time step *k*, $w_r(k)$ is the process noise assumed to be white Gaussian with zero mean, and its covariance matrix is denoted as *Q*. The function *f* and *F* depends on the robot model.

Prediction of the state covariance matrix $P(k+1|k)$ is given by

4 $$\begin{aligned} P(k+1|k) = \nabla F_X P(k|k) \nabla F_X^T + Q \end{aligned}$$

where $\nabla F_X$ is the Jacobian of *F* with respect to *X* evaluated at *X*(*k*|*k*), *P*(*k*|*k*) denotes the state covariance matrix at time step *k*.

The observations $Z(k+1|k)$ that are obtained from the state $X(k+1|k)$ at the time step *k* are given by

5 $$\begin{aligned} Z(k+1|k) = H(X(k+1|k))+v(k) \end{aligned}$$

where *H* defines the nonlinear coordinates transformation from the state to the observation $Z(k+1|k)$. The observation noise *v*(*k*) is assumed to be white Gaussian with zero mean and uncorrelated the process noise $w_r(k)$. In observation process, the $Z(k+1|k+1)$ is measured from the actual environment, and its covariance matrix is denoted by $R(k+1|k+1)$. To compare observations in sequential time steps, the kidnapping can be detected and distinguished.

Update the state and the associated covariance matrix using the observation $Z(k+1|k+1)$,

6 $$\begin{aligned} \begin{aligned} X(k+1|k+1)&= X(k+1|k)+K(k+1)[Z(k+1|k+1)\\&-H(X(k+1|k))]\\ P(k+1|k+1)&= P(k+1|k)\\&-K(k+1)S(k+1)K(k+1)^T \end{aligned} \end{aligned}$$

where

7 $$\begin{aligned} \begin{aligned} K(k+1)&= P(k+1|k)\nabla H_X^T S(k+1)^{-1}\\ S(k+1)&= \nabla H_X P(k+1|k)\nabla H_X^T+R(k+1|k+1) \end{aligned} \end{aligned}$$

and $\nabla H_X$ is the Jacobian of *H* with respect to *X* evaluated at $X(k+1|k)$.

The main structure and workflow of P-DGKD are same with DGKD. The main difference with DGKD is the metrics. In P-DGKD, the uncertainty of state is combined to the metrics. New improved metrics have the same name as DGKD and are used in original processes separately.

With the root-mean-square, $Q_p$ is given by

8 $$\begin{aligned} \begin{aligned} Q_p&= \sqrt{\frac{1}{N} \sum _{i=1}^{N} V_i(k)^T S_i(k)^{-1} V_i(k)} \end{aligned} \end{aligned}$$

where

9 $$\begin{aligned} \begin{aligned} V_i(k)&= Z_i(k+1|k+1)-Z_i(k+1|k)\\ S_i(k)&= R_i(k+1|k+1)+R_i(k+1|k)\\ \end{aligned} \end{aligned}$$

and *N* represents the number of the overlapped observed features between sequential time step *k* and $k+1$. $Z_i$ denotes the observation of $i_{th}$ overlapped feature. $Q_o$ is given by

10 $$\begin{aligned} \begin{aligned} Q_o&= \sqrt{\frac{1}{N} \sum _{i=1}^{N} W_i(k)^T M_i(k)^{-1} W_i(k)} \end{aligned} \end{aligned}$$

where

11 $$\begin{aligned} \begin{aligned} W_i(k)&= Z_i(k+1|k+1)-Z_i(k|k)\\ M_i(k)&= R_i(k+1|k+1)+R_i(k|k)\\ \end{aligned} \end{aligned}$$

$Q_s$ is given by

12 $$\begin{aligned} \begin{aligned} Q_s&= \sqrt{\frac{1}{M} \sum _{i=1}^{M} D_i(k)^T O_i(k)^{-1} D_i(k)} \end{aligned} \end{aligned}$$

where

13 $$\begin{aligned} \begin{aligned} D_{mi}(k)&= X_{mi}(k+1|k+1)-X_{mi}(k+1|k)\\ O_{mi}(k)&= P_{mi}(k+1|k+1)+P_{mi}(k|k)\\ \end{aligned} \end{aligned}$$

and *M* denotes the number of the overlapped features between sequential time step *k* and $k+1$.

Comparing with DGKD, P-DGKD adds the associated covariance matrix into metrics. This modification can decrease the effect of the increasing uncertainty in SLAM process. It can decrease the rate of false alarm in the whole detection reports. It causes that the whole performance of P-DGKD is better than DGKD. About the thresholds of metrics, the method proposed in DGKD is used in P-DGKD. Detection and classification condition are the same as that in DGKD.

## Simulation results

Simulations were conducted to investigate the feasibility and accuracy of the proposed method. We implemented the described method using MATLAB in a personal computer (CPU: 3.40GHz Intel Core i5, Memory: 8 GB DDR3). The source code is based on EKF-SLAM in SLAM package of Tim Bailey. We modified and added our method in it. Other basic parameters for simulations are same to our previous research of DGKD [9]. The map of simulation has been shown in Fig. 5a. We did simulations in a no actual kidnapping situation during the whole SLAM process. The results of metrics of $Q_p$ and $Q_s$ are shown in Fig. 7.

In non-kidnapping situation, the value of metric $Q_p$ is less than the first threshold $T_{p1}$, and metric $Q_s$ is lower than the threshold $T_s$. Comparing with the simulation results of DGKD shown in Fig. 6, the value of both metrics is below the thresholds after time steps increased. This means that, although the uncertainty of the state increased by the SLAM process, influence in kidnapping detection has been decreased.

For getting the whole performance of P-DGKD, we also did tests in kidnapping situation during SLAM process. The kidnapping is a man-made movement to the robot to simulate the real kidnapping that the robot is moved by the human in the environment. Then, we can judge whether the report of P-DGKD is a true alarm or a false alarm or no alarm. The result of the metrics where kidnapping happened at time step 600 is shown in Fig. 8.

Before the kidnapping, the value of metric $Q_p$ is lower than the first threshold $T_{p1}$. When kidnapping happens in the 600th time step, $Q_p$ is larger than the second threshold $T_{p2}$. Moreover, the value of the metric $Q_s$ also exceeds the threshold $T_s$. It means P-DGKD can detect kidnapping correctly in this situation.

Several other simulations were conducted to show the performance of P-DGKD and DGKD. Different simulations were conducted to verify the performance under the kidnapping situation and the non-kidnapping situation. In each simulation, one-time kidnapping event is set randomly from step 500 to time step 800. The results processed by ROC are shown in Table 2. About report of kidnapping in DGKD and P-DGKD, the true-positive rate is the fraction of detected kidnapping out of the total number of actual kidnapping events, and the false-positive rate is the fraction of non-kidnapping time steps that is incorrectly detected out of the total number of actual non-kidnapping time steps. Comparing with DGKD, the false-positive rate is decreased in P-DGKD, which means that the possibility of the false alarm is decreased. At the same time, the true-positive rate of P-DGKD is also decreased a little comparing with DGKD. That means although the rate of false alarm is decreased, P-DGKD is not as sensitive as DGKD dealing with the real kidnapping. Totally considering, the performance of P-DGKD is better than DGKD.

**Table 2:** Operating characteristics of detection

Detection	True-positive rate	False-positive rate
DGKD	0.9900	0.1203
P-DGKD	0.9800	0.0431

## Conclusion

In this paper, we have presented a probabilistic double guarantee kidnapping detection in SLAM. Compared with our proposed method DGKD, P-DGKD is more suitable for the large-scale environment. In P-DGKD, a probabilistic formulation is used to add uncertainty of robot posture and features in the metrics which can judge whether kidnapping happened. Using the proposed method, the kidnapping event can be detected accurately and robustly. Simulation result shows the validity and feasibility of the proposed method.

In future studies, we plan to detect the kidnapping of the robot in the case that the kidnapping happens over a long time. Our proposed method can well solve the problem for a short-time kidnapping event. However, if the kidnapping happens in a long time, such as a human carrying the robot for a long distance, or the robot slips all the time in a specific area, the method introduced in this paper could result in a failure. Further verification of DGKD in a real environment is also our future work.
